# Robotic staging procedure performed using medtronic hugo ™ RAS in an endometrial cancer patient: A case report

**DOI:** 10.1016/j.heliyon.2023.e23756

**Published:** 2023-12-16

**Authors:** Kim-Seng Law

**Affiliations:** a Department of Post-Baccalaureate Medicine, National Chung Hsing University, Taichung, Taiwan; b Department of Obstetrics and Gynecology Tung's Taichung Metroharbor Hospital, Taichung, Taiwan

**Keywords:** Case report, Endometrial cancer, Hugo ™ RAS, Robotic surgery

## Abstract

We describe the first ever reported staging procedure using the new robotic system Hugo ™ RAS for an early endometrial cancer patient with uneventful perioperative outcomes.

## 1 Introduction

Endometrial cancer is the most prevalent gynecologic cancer in developed countries and its standard treatment is surgery, in particular minimal invasive surgery [1]. Da Vinci surgery (Intuitive surgical Sunnyvale, CA) has been widely used in the past decades with its precision, magnified surgical field, faster recovery and short hospital stay with surgical and oncological results comparable to conventional laparoscopy [2]. A new robotic surgical devices have been recently approved by the CE with “open console”, flexibility of its four separate arms carts with dozen cases have been successfully performed by urologist and proctologist [[2], [3], [4], [5]]. We herein describe the first case reported in the English literature about the staging procedure in an early endometrial cancer performed by the Medtronic Hugo ™ RAS by exploring its feasibility, console time, operative time as well as surgical outcomes.

## 2 Case report

A 64-year-old G1P1 woman without any systemic diseases or comorbidities and previous abdominal surgery, with BMI of 21.48, and a history of CIN2 who underwent conization 6 months ago presented with postmenopausal bleeding at our clinic. Transcervical resectoscope (TCR) with the suspicious of endometrial lesion was arranged and the pathology reveals an endometroid endometrial cancer grade 3. Preoperative CT scan reveal no retroperitoneal lymphadenopathy and no visible residual tumor in the cavity.

**Fig. 1:**
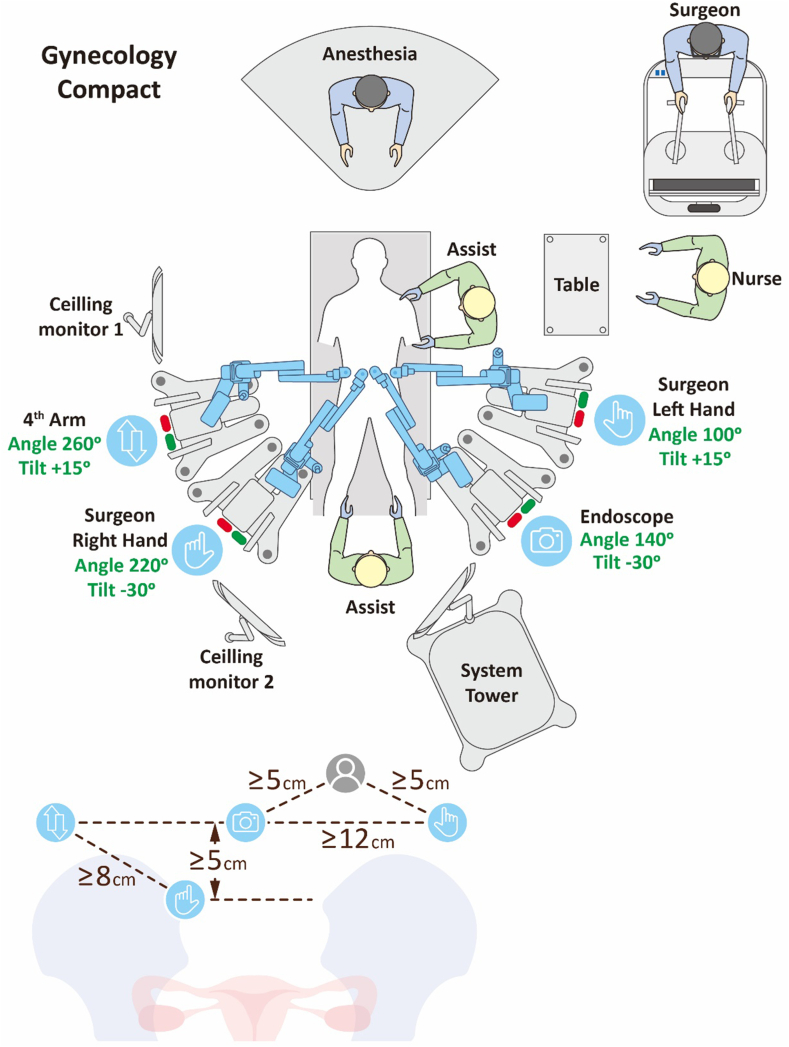
The compact configuration for the four arm carts with the bipolar fenestrate grasper over the left arm, monopolar hot shear on the right arm and Cadiere forcep on the fourth or reserve arm.

Preoperative carts setup was planned as depicted in Fig. 1 with Arm 1 setting at 100° with tilt +15° equipped with bipolar fenestrated grasper and the endoscopy(Arm 2) at 140° tilt −30°, third arm equipped with monopolar curved shears with 220° tilt −30° and fourth arm with 260° tilt +15° equipped with Cadiere forceps (Fig. 1). The distance between relative arms and endoscopy and the assistant arm was also depicted in Fig. 1 with at least 8cm between them.

**Fig. 2:**
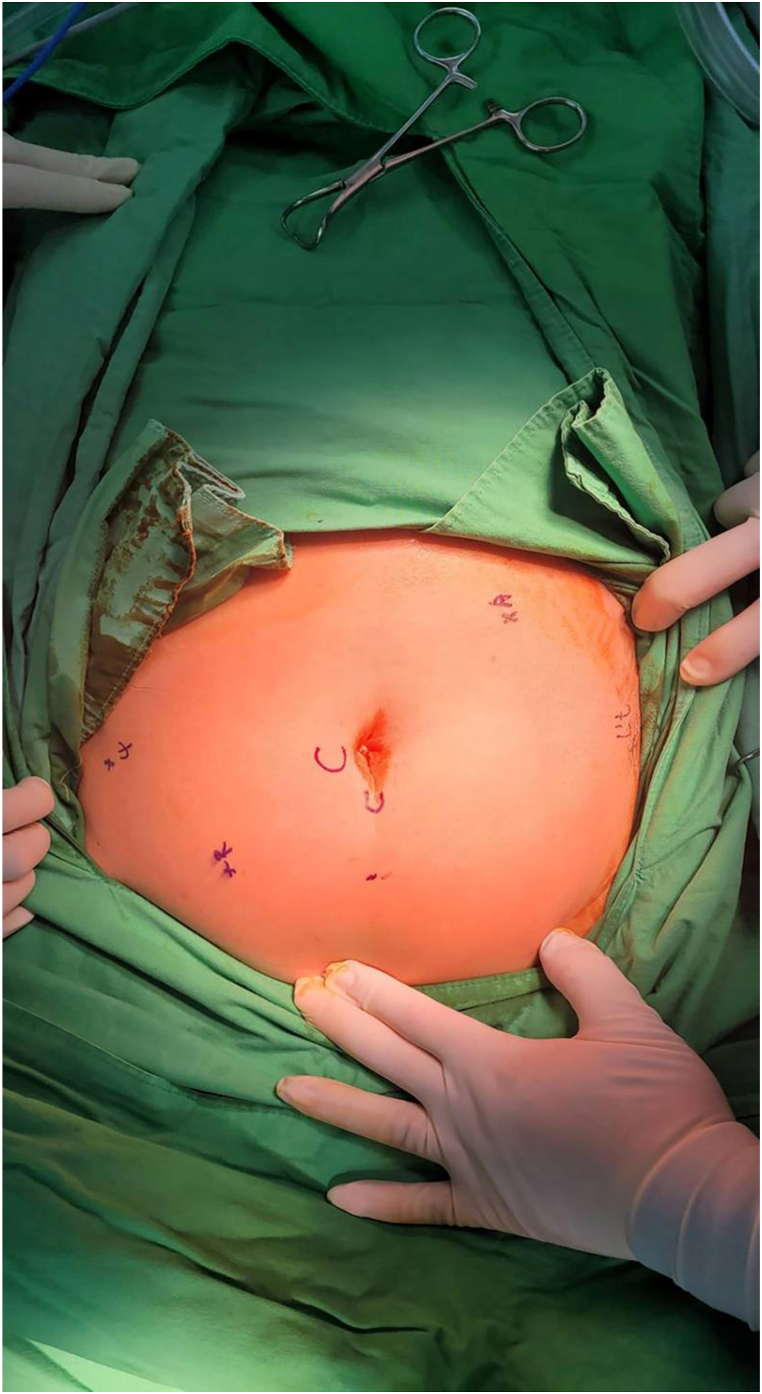
Ports placement for the procedure (R: Right Arm; L't: Left Arm; 4: Fourth Arm; A: Assistant port).

Ports placement was planned and is shown in Fig. 2.

**Fig. 3:**
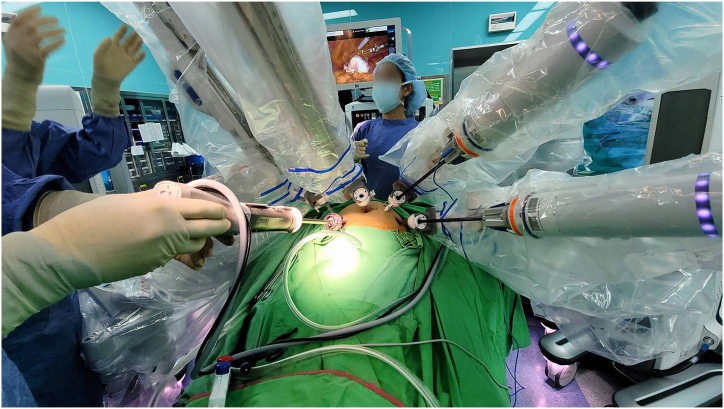
Versatile application of the endoscopy through the assistant port with direct visual -guided entry of others instrumentation apart from staying put in the endoscopy cart only.

All the instrument entry was visual -guided with the endoscopy either through the endoscopic arms or more flexibly via the assistant port (Fig. 3) A 11mm camera port (arm 2) with three 8mm instrument's ports and a 11mm assistant ports were made.

The flexible four arm carts needed to be planned before docking with both tilting and horizontal angles known to the whole surgical teams and adjust accordingly during the procedure to reduced collision of the arms and for more easily accessible to targets.

**Fig. 4:**
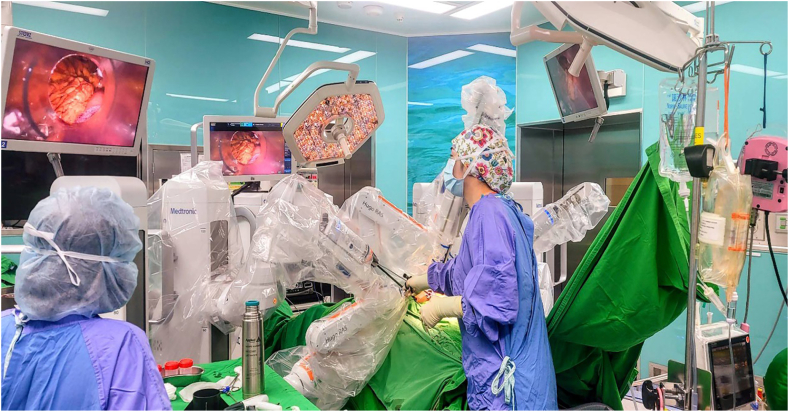
The Palmer's position of the assistant enables the procedure to be conducted smoothly with a long instrument.

The procedure carried out was total hysterectomy and bilateral salpingooophrectomy with bilateral tubes cauterized first with bipolar grasper to prevent tumor spillage during the procedure. Right pelvic lymphadenectomy was performed systemically followed with paraaortic lymphadenectomy and left pelvic lymphadenectomy. Modification of arms 3 (monopolar shear) was made with tilting angle changing from −30 to −15 for better access to upper paraaortic area during the procedure. All the lymph nodes were inserted in three separate endobags and extracted through the 11mm assistant port. The assistant port was made through the Palmer's point with a long instrument for a better assistance (Fig. 4). The uterus and adnexae were extracted via vaginal and cuffs suture with 1-0 coated Vicryl intracorporeally. Total blood loss of 50 cc were estimated with docking and console time of 10 and 215 mins, respectively.

Post operative recovery was smooth with patient discharge on day 4. The final pathology reveals stage 1a grade 3 endometroid adenocarcinomas with a total of 17 lymph nodes extracted with all of them negative and no lymph vascular space invasion was noted on the specimens. Post operative adjuvant radiotherapy was planned per protocol of NCCN Guidelines Version 1.2024.

## 3 Discussion

No gynecologic oncology case using the newly launched robotic device (Hugo ™ RAS) has been reported thus far. We demonstrated the feasibility as well as perioperative outcomes in an endometrial cancer staging procedure by using this new robotic platform.

**Fig. 5:**
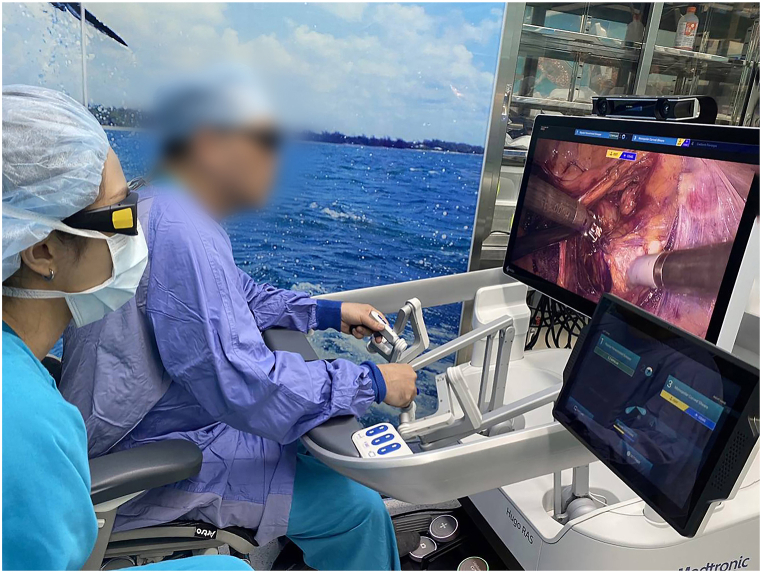
Open surgical consoles enable the surgeon to communicate with the whole team's member and instructive to the observer during the whole procedure.

With an“open” surgical console and an HD–3D passive display, the surgeon not only assure a comfortable and ergonomic sitting posture during the whole procedure, but it can also be more instructive for others who want to observe the procedure simultaneously and the benefits of open communication between the surgical teams (Fig. 5). Flexibility and applicability of the four arms is clearly demonstrated in this case indeed Slight modification of the tilting angle during upper abdominal approach enable the procedure to be conducted smoothly without adjusting the surgical booth or even redocking and the four arms carts could be precisely placed at different sites with the use of the default laser measurement unit and more flexible approach of the surgical instruments due to the versatility of joints of the arms cart.

Adjustment of the new setting for surgeon are anticipated with some “lagging” time noted while shifting between the bipolar and unipolar instrument as well as changing between the reserve arm and the third arm with the clutch side pedal. The availability of more instrument option is another issues that should be addressed as compared to the da Vinci system in which the Hugo RAS lacking a vessel sealer and more delicate tissue handling instruments However, it did not limit its feasibility in a complicated procedure like this. Another disadvantage of the new platform is its lack of “firefly” device as installed in the da Vinci Xi system while performing sentinel lymph node in early-stage endometrial cancer and systematic lymph node dissection would be anticipated in such cases.

When para-aortic/upper abdominal procedures were performed, we experienced limited motion of the robotic arms 2 and 3. This limitation was overcome by increasing the negative tilt of arm3 from −30 to −15 as suggest by Alletti et al. [6]. With the setting of “compact “configuration with narrow spaces between the four carts, there are cumbersome encountered during the procedure with the assistant having to go through the left Palmar's point to reach the targets and collision may sometimes note. A modified butterfly configuration with wider spaces between the carts will therefore better for a smoother procedure.

Shifting from conventional da Vinci system to a novel separated four arms carts and an open console needed accreditation with a full 48 hour course training by a knowledgeable proctoreven in a previous skillful robotic surgeon. A dry lab or cadaveric or animal hand-on practice made the procedure smooth.

Robotic surgery has been demonstrated safe and feasible in elderly patients without compromising the effectiveness and increasing the morbidities [7,8]. Careful selection of frail elderly patients undergoing robotic surgery should be excised as previous study has demonstrated more complications in this cohorts [9].

We demonstrated the feasibility of the new Hugo ™ RAS robotic system in an endometrial cancer staging procedure and anticipated shorter docking and console time with cases accumulating in the future.

## Ethics statement

The study was approved by Tung's Taichung Metroharbor Hospital Institutional Review Board, IRB # 112024, and written informed consent was obtained from the patient.

## Data availability statement

Data will be made available on request.

## CRediT authorship contribution statement

**Kim-Seng Law:** Conceptualization, Data curation, Formal analysis, Writing – original draft, Writing – review & editing, Methodology.

## Declaration of competing interest

The authors declare that they have no known competing financial interests or personal relationships that could have appeared to influence the work reported in this paper.
